# Dataset creation of thermal images of pomegranate for internal defect detection

**DOI:** 10.1016/j.dib.2025.111538

**Published:** 2025-04-03

**Authors:** Ashvini Gaikwad, Manoj Deshpande, Varsha Bhole

**Affiliations:** A. C. Patil College of Engineering Kharghar, Navi Mumbai, Maharashtra, India

**Keywords:** Thermal imaging, Defect detection, Pomegranate dataset, K-mean segmentation

## Abstract

Datasets are crucial in various fields, especially in the context of machine learning, data science and research. Datasets are used to train machine learning models. A model learns patterns and relationships from the data it is exposed to. The dataset used for training a machine learning model shall be diversified and consist sufficient samples of desired categories. This paper presents various steps and its outcome in preparing the dataset of digital and thermal images of pomegranate for recognising internal defects. The defects in fruits are often categorised as surface defects and internal defects. The surface defects are recognised with digital RGB image but fails to give insight about the internal structure of the fruit in which we are often interested. The thermal images can be used to detect the internal defects in fruits. When a fruit is subjected to temperature difference as compared to the surrounding, the thermal emissions from fruit captured through a thermal camera (thermal image) gives the key information about the internal damages in the fruit. The internal defects are reflected in thermal image as variations in temperature of adjacent pixels. The k-mean segmentation is applied for identifying internal defects with thermal images in pomegranates to categorize them viz. No defect, major defect and minor defect. This information is useful for training a machine learning algorithms that are intended for bulk processing in the field of fruit defect detection and classification.

Specifications of the camera.

**Table:** 

Subject	Fruit defect detection, thermal imaging
Specific subject area	Internal defect detection and quality prediction
Type of data	Thermal images of fruits
Data collection	The thermal images are captured through Seek Thermal (Android compatible) camera with following specifications 206 × 156 Thermal Sensor, 36°Field of View, < 9 Hz Frame Rate, Focusable Lens, −40F° to 626°F Detection Range. The data was collected between January 2024 to May 2024 Location: A.C. Patil College of Engineering Kharghar Navi Mumbai, Maharashtra, India.
Data format	Raw jpg
Data accessibility	Repository Name: Mendeley Data URL to access data https://data.mendeley.com/datasets/djcgvgtcfm/1 doi: 10.17632/djcgvgtcfm.1

## 1 Value of Data

• The fruits quality is dependent on many parameter and it happens that a fruit appears good from surface but may have spoilage inside.
• Hence there has to be a mechanism for knowing the internal structure of fruits and vegetables. This internal structure knowledge is obtained through thermal imaging.
• Every object has unique thermal characteristics on account of which it radiates energy to the surrounding or captures thermal energy from surrounding at a uniform rate.
• The thermal images gives the information about temperature of each point (pixel) of the object. The defects are noted when the thermal characteristics of adjacent pixels differ from each other.
• The data of thermal images is useful for training and implementation of a machine learning model in post-harvest material handling industry which processes fruits and vegetables in large quantum. This is particularly useful for food processing industry which segregates good and bad fruits. The image processing based techniques can be deployed in such cases to avoid manual operating errors and hence assure best quality of service.

## 2 Background

Considering the horticulture scenario, India is the net exporter of fruits and vegetables. India exports mango, grapes, banana, pomegranate and oranges. Climate change has been perceived as threat and will have impact on horticultural crops, due to erratic rainfall, more demands for water and enhanced biotic and abiotic stresses. This develops defects in fruits on the surface as well as inside. The surface defects are visible and such fruits can be sorted through visual inspections as well as digital RGB image processing based system [1]. However, fruits with internal damages may look good on the surface but can lead to spoilage of entire batch [[4], [5]]. Hence an automated and bulk processing systems for fruit defect detection shall be integrated to analyse the surface and internal defects [6]. The digital and thermal image processing techniques based on AI techniques, machine learning and deep learning requires training with appropriate dataset [[2], [3]].

A dataset is crucial for training a machine learning model for fruit defect detection [[7], [8]]. It provides the necessary examples for the algorithm to learn patterns and distinguish between healthy and defective fruits. A diverse dataset helps to train the model for different defects and variations in fruits, improving its accuracy and reliability in real-world scenario [9]. Hence to create a dataset of pomegranate for internal defect detection the RGB digital image and thermal image of the pomegranate is taken. Using thermal images fruits are categorised in three parts as no defect, minor defect and major defect [[10], [11]].

## 3 Data Description

The dataset consists of thermal images of pomegranate taken post harvesting. The pomegranates are at first stored and ensured that they are brought to temperature below the ambience. The temperature difference is necessary to allow the thermal radiation between pomegranate and the atmosphere. The digital image and thermal image of the same fruit is taken by placing both cameras at same angle. The fruits with digital and thermal images are categorized in three parts viz. no defect, minor defect and major defect. These categorization is done manually. The samples of digital and thermal images are mentioned in Table 1.

**Table 1:** Sample dataset images of pomegranate.

## 4 Experimental Design, Material and Methods

For creating the dataset, the specifications of cameras used for capturing digital and thermal images are mentioned in specification table. The digital and thermal image for each sample of the pomegranate is captured and a total of 1486 images are acquired categorized in three parts. The fruit sample was cut to validate the internal defect seen through the thermal image. The heat transfer properties of fruit can be influenced by internal issues like bruises, rot or diseases. Consequently, the areas affected by these problems shows distinct thermal characteristics in comparison to the healthy regions. The steps followed while taking photographs are -

• Refrigerate pomegranate for 60 min.
• Take out refrigerated pomegranates and keep it at room temperature for 10 min.
• Place the pomegranate on the table and click digital image, thermal image with full spectrum and black & white thermal image.

**Fig. 1:**
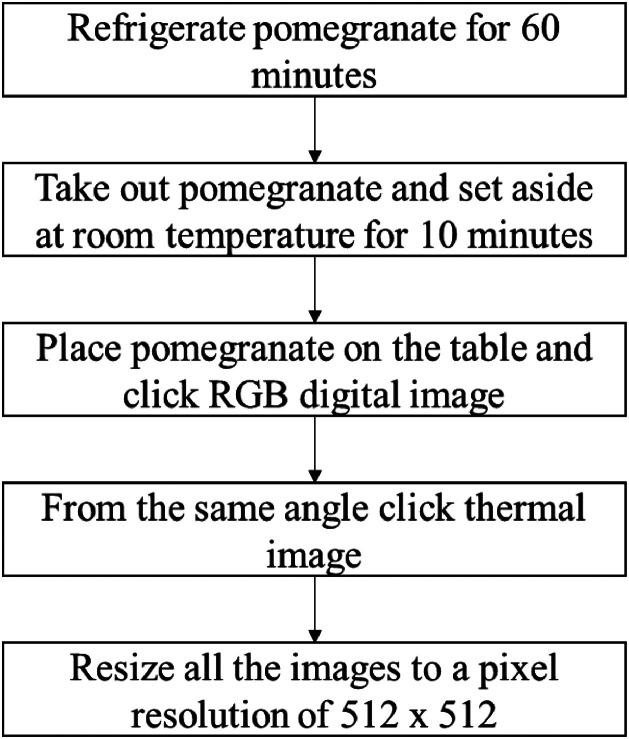
Workflow of capturing images for dataset creation.

**Fig. 2:**
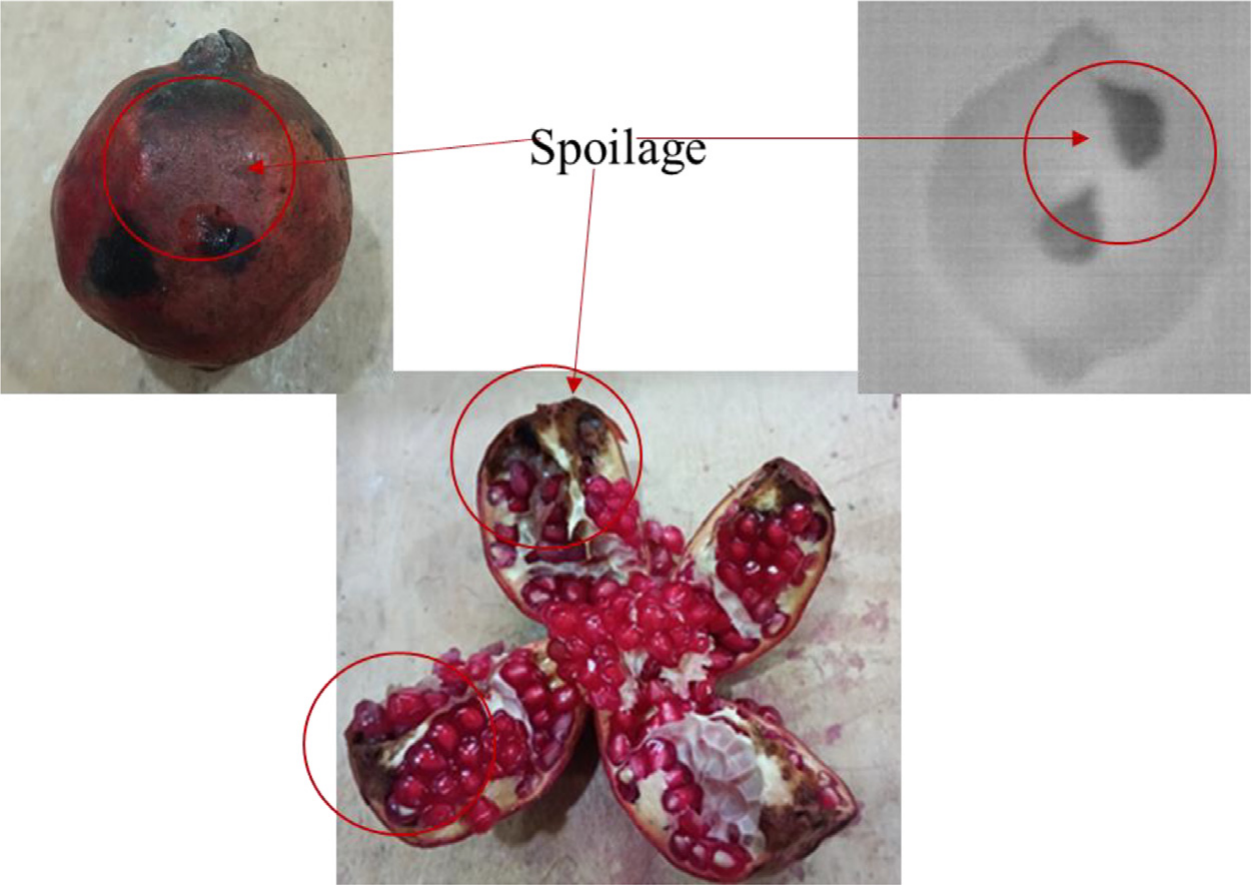
Validation of rot or spoilage by cutting pomegranate.

The workflow for dataset preparation is shown in Fig. 1. The workflow starts with incubating the pomegranates for 60 minutes. The incubation is necessary to create the temperature difference between the pomegranate and surrounding atmosphere so that the thermal images can be captured correctly. The images captured from digital and thermal camera are resized for 512 × 512 pixels to maintain the uniformity among the images. The resizing helps to improve the computational efficiency of the system, reduce over fitting, enhance memory usage and training speed [12,13]. The pomegranate images are categorized in three parts as major defect, minor defect and no defect on the basis of manual visual inspection. The sample RGB and thermal images in three categories of no defect, minor defect and major defect are shown in Table 1. To validate the internal defects like rot and spoilage, the pomegranate fruits were cut to observe the internal details. A sample of internal spoilage reflected on the thermal image is shown in Fig. 2.

**Fig. 3:**
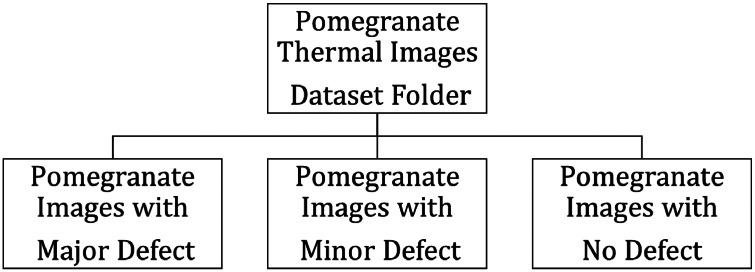
Structure of the dataset.

The dataset of digital images of pomegranate is discussed in [16]. This dataset of digital images is helpful for identifying skin defects or outer defects in pomegranates. However, internal spoilage defects are beyond the scope of digital images. The thermal images helps in identifying the internal defects and spoilage. The structure of the dataset is shown in Fig. 3. The pomegranate images in the dataset are categorized in three folders as pomegranate images with major defect, pomegranate images with minor defect and pomegranate images with no defect. Each folder consists of digital and thermal images in respective categories.

## 4.1 Defect detection with segmentation

Segmentation is first process in detecting defects. It is partitioning a digital image into multiple segments (sets of pixels) which helps to simplify or change the representation of an image into something more meaningful and easier to analyse. Thermal images capture temperature variations, but they lack the rich detail of visible light images. Segmentation helps separate objects and regions of interest from the background. This allows you to focus on specific areas for further analysis. The segmentation of thermal images allows quantifying temperature within the area of interest. Hence it creates a clearer and informative visualization of the thermal data.

**Fig. 4:**
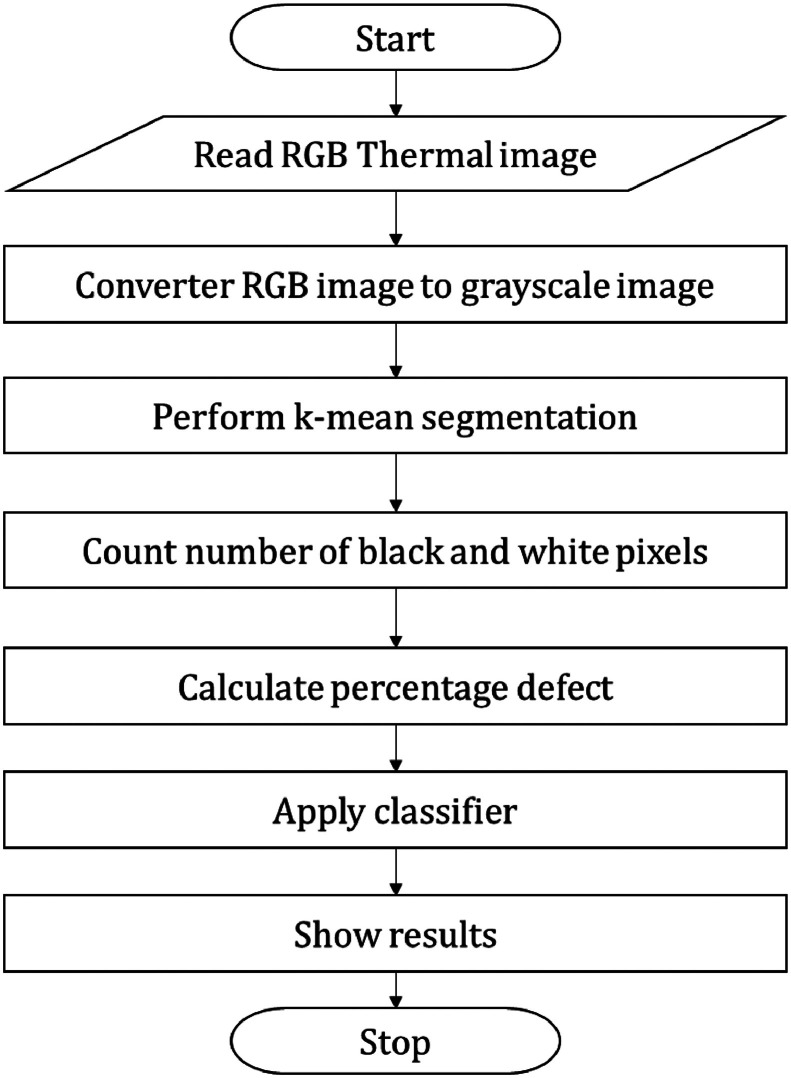
Defect detection work flow.

The images are segmented using k-mean segmentation. K Means is a clustering algorithm. Clustering algorithms are unsupervised algorithms. It is used to separate distinct classes or clusters in the provided data according to the degree of similarity between the data. Fig. 4 shows methodology adopted for segmentation. The MATLAB software with image processing toolbox is used for programming the code. For segmentation of pomegranate images, the threshold level is set at 0.45. The RGB thermal image is first converted into grayscale image and then it is segmented and converted into a binary image with appropriate threshold for detecting the black spots which are indicative of the defect in the fruit.

The configuration settings for k-mean segmentation are mentioned in Table 2. The configuration settings include number of clusters, distance metric, iterations, pixel features and mask.

**Table 2:** Configuration settings of k-mean segmentation.

Particulars	Value
Number of clusters	2
Distance metric	Squared Euclidean Distance
No. of iterations	3
Pixel features	Grayscale (0.45)
Mask	1 (defect mask)

The classifier is used to classify the fruit in the categories of major defect, minor defect and no defect. For no defect, the defect contribution less than 10 % is considered. The defect percentage between 10 and 25 % is considered as minor defect and the defect contribution more than 25% is considered as major defect. The results on sample basis in each category is presented in Table 3.

**Table 3:** Results of defect detection of pomegranate.

This dataset of thermal images of pomegranates provides a valuable resource for the development and evaluation of computer vision algorithms for various applications of quality control and sorting. The dataset includes thermal images of pomegranate in various defect categories, offering a diverse and representative sample for robust model training and testing [[14], [15]]. Conclusively, this dataset aims to aid (image processing based) developments in the field of post harvesting field for improving efficiency and sustainability of supply chain.

## Limitation

This dataset is limited for pomegranate fruit. The results of image processing based experimentation on this fruit may not be applicable on other fruits

## Ethics Statement

This dataset is made available in public. This data can be used with proper citation. No animals were involved and no food was wasted during this experimentation.

## CRediT Author Statement

**Ashvini Gaikwad:** digital and thermal photographs, dataset creation**,** classification of images, initial paper drafting, **Manoj Deshpande**: Segmentation and defect detection, validation **Varsha Bhole**: Validation and editing

## Data Availability

Mendeley DataPomegranate Thermal Images (Original data)
